# Pharmacokinetics and pharmacodynamics of growth hormone in patients on chronic haemodialysis compared with matched healthy subjects: an open, nonrandomized, parallel-group trial

**DOI:** 10.1111/j.1365-2265.2007.02962.x

**Published:** 2007-11-01

**Authors:** Irene H Langbakke, Jakob N Nielsen, Mia P Skettrup, Angela Harper, Thomas Klitgaard, Angelika Weil, Eva Engelhardt, Martin Lange

**Affiliations:** * Departments of Clinical Pharmacology Bagsvaerd, Denmark; † Departments of Biostatistics Bagsvaerd, Denmark; ‡ Departments of Clinical Reporting Bagsvaerd, Denmark; § Departments of Biomodelling, Novo Nordisk A/S Bagsvaerd, Denmark; ¶ APEX Research Munich, Germany; ** Clinical Research, Novo Nordisk Inc New Jersey, USA

## Abstract

**Background:**

GH may be beneficial in treating patients with end-stage renal disease (ESRD). However, the efficacy and safety of GH could be compromised by the potential for accumulation in the circulation.

**Objective:**

The objective was to investigate the pharmacokinetics and safety of GH treatment in ESRD patients.

**Design:**

This was an open, nonrandomized, single-centre parallel-group study lasting 8–9 days.

**Subjects:**

Eleven adult ESRD patients and 10 matched healthy individuals received recombinant human GH (50 µg/kg/day for 7 days) by subcutaneous injection; there were two dose reductions (25%) from Day 5/7. ESRD patients underwent dialysis four times.

**Measurements:**

Serum concentrations of GH, insulin-like growth factor-I (IGF-I), insulin-like growth factor binding protein-I (IGFBP-I), IGFBP-III and GHBP were measured. The primary end-point was GH exposure [area-under-the-curve (AUC) calculated from the 24-h profile] on Days 7–8.

**Results:**

GH AUC_0–24 h_ was greater for patients (387·91 ± 134·13 µg h/l) than healthy subjects (225·35 ± 59·63 µg h/l) and the 90% confidence interval (CI) for the estimated patient : healthy subject ratio (1·40–2·07) was not within the acceptance interval (0·67–1·50). GH AUC_18–24 h_ for patients and healthy subjects (3·03 ± 2·71 µg h/l and 6·37 ± 4·21 µg h/l) returned approximately to baseline (2·86 ± 3·91 µg h/l and 1·09 ± 1·43 µg h/l); terminal half-life (*t*_1/2,z_) was shorter for patients (2·28 ± 00·43 h *vs.* 3·23 ± 00·75 h). No major safety issues were identified.

**Conclusions:**

Results demonstrate a difference between patients and healthy subjects regarding GH AUC_0–24 h_. However, GH concentrations for both groups were comparable to baseline by 20–22 h, thus GH was not retained in the circulation of ESRD patients.

## Introduction

Patients with end-stage renal disease undergoing haemodialysis commonly suffer from malnutrition, which represents one of the main clinical problems for these patients.^1^ The prevalence of malnutrition in dialysis patients is reported to range from 30% to 50% or more.^2^,^3^ The state of malnutrition and the malnutrition-induced systemic inflammation in patients with end-stage renal disease (ESRD) have been associated with the increased mortality and morbidity observed in these patients.^4^–^9^ The cause of the poor nutritional status is multifactorial, and is associated with anorexia and reduced nutrient intake,^3^,^10^,^11^ metabolic and hormonal alterations,^12^ and catabolic effects associated with dialysis.^10^ Although as yet unproven, treatment of malnutrition in these patients can potentially improve clinical outcome and reduce the risk for morbidity and mortality. Thus, a number of preventive and therapeutic measures have been used to treat malnutrition in patients with ESRD, including anabolic hormones such as GH.

The potential beneficial effects of recombinant human GH (rhGH) in the treatment of patients with ESRD have been demonstrated both in short-term studies and long-term (up to 6 months) randomized, controlled trials. Treatment with rhGH has been shown to reduce protein catabolism,^13^,^14^ increase muscle area and strength,^14^–^18^ increase erythropoietin synthesis,^19^ and to improve inflammation status and quality of life.^20^ A recent 6-month study showed a positive effect of GH treatment on lean body mass and cardiovascular risk factors.^21^ Furthermore, GH administration is associated with improvements in several nutritional parameters, such as increases in serum insulin-like growth factor-I (IGF-I),^13^,^14^,^22^ serum albumin^14^ and transferrin,^13^ and a reduction in blood urea nitrogen.^13^

rhGH has become available for the treatment of children with chronic renal failure and has been shown to be safe and efficacious. However, the physiological response to exogenous GH treatment in adults may be different compared to that in children. As GH is cleared partly by the kidneys in healthy subjects,^23^–^25^ investigation of a possible influence of renal impairment on drug elimination would be relevant.^23^,^25^,^26^ The primary aim of the current trial was thus to investigate the pharmacokinetic (PK) aspects and safety of treatment with rhGH (daily subcutaneous injection) in patients with ESRD compared with those in healthy subjects. A secondary objective was to investigate the pharmacodynamic (PD) responses to GH administration and to compare these in patients with ESRD and in healthy subjects.

## Subjects and methods

### Subjects

Twenty-two subjects (11 ESRD patients, 11 healthy subjects) were enrolled in the study and 21 subjects (mean age 51 years, range 24–66 years) received treatment with rhGH. There was one screening failure. One healthy subject withdrew consent prior to treatment and was withdrawn from the study. The study was performed at APEX Research Centre (Munich, Germany) in accordance with the Declaration of Helsinki^27^ and International Conference on Harmonization Good Clinical Practice^28^ and was approved by local Ethics Committee. All subjects gave written informed consent. Subject demographics are presented in Table 1.

**Table 1:** Baseline characteristics of patients and healthy subjects

Variable	Patients (*N* = 11)	Healthy subjects (*N* = 10)
Age (years)	51·3 ± 13·5 (24–66)	50·2 ± 12·5 (28–64)
Sex, *N* (%)		
Female	3 (27)	3 (30)
Male	8 (73)	7 (70)
Race, *N* (%)		
White	11 (100)	10 (100)
Height (cm)	176·5 ± 7·0 (163–189)	172·5 ± 6·8 (162–182)
Weight (kg)	77·5 ± 8·3 (62·2–92·1)	78·3 ± 7·5 (63·5–90·8)
BMI (kg/m^2^)	25·0 ± 3·2 (20·3–29·2)	26·3 ± 1·8 (22·8–28·8)
Time in present HD treatment (years)	2·1 ± 2·2* (0·3–6·7)	N/A
Kt/V	1·4 ± 0·3 (1·2–2·1)	N/A
Systolic blood pressure	127·7 ± 34·0	129·5 ± 10·3
Diastolic blood pressure	80·2 ± 9·3	81·3 ± 7·6
Fasting plasma glucose (mmol/l)	4·6 ± 0·5	4·8 ± 0·5

Values are the mean ± SD (range), except for sex and race, *N*(%).

* Based on 10 patients.

BMI, body mass index; HD, haemodialysis; N/A: not applicable.

Inclusion criteria for patients were male or female; age ≥ 18 years; and in chronic and stable haemodialysis 3 months prior to enrolment (measure of dialysis efficiency Kt/V > 1·2 and/or haemodialysis performed for 4 h, three times weekly). The healthy control population was chosen to enable a valid comparison with ESRD patients. Main exclusion criteria for all subjects were active malignant disease; diabetes (fasting blood glucose 7·0 mmol/l); critical illness (need for respiratory or circulatory support); parathyroid hormone 500 pmol/l; chronic treatment with steroids; treatment with immunosuppressive agents; active vasculitis; heart failure (New York Heart Association class III–IV); severe hepatic disease; and severe chronic systemic inflammation.

### Study design

**Fig. 1:**
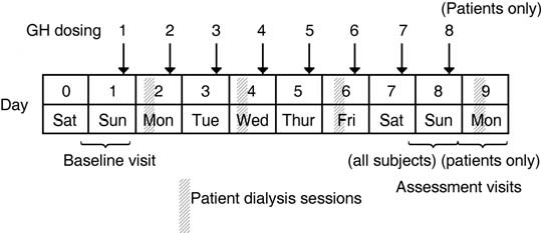
Trial design. All subjects had eight visits to the trial centre, seven daily doses of rhGH and a follow-up visit (Days 16–19). Patients had an additional assessment visit on Days 8–9, received an additional rhGH dose on Day 8 and had four dialysis sessions over the 9-day period.

This was an open, nonrandomized, parallel-group study, comprising eight visits for healthy subjects and nine visits for patients with ESRD. The following visits were included for all subjects (Fig. 1): screening visit, baseline visit (Days 0–1), outpatient visits (Days 2–6), assessment visit (Days 7–8) and a follow-up visit (Days 16–19). Patients with ESRD received haemodialysis treatment on alternate days three times during the study, and had an additional assessment visit (Days 8–9), which included a dialysis session. The first dose of rhGH (Norditropin® SimpleXx®, Novo Nordisk A/S, Bagsvaerd, Denmark) was administered in the evening of Day 1 and daily dosing continued up to Day 7. Patients received an additional dose on Day 8 in order to record the GH exposure during dialysis. All subjects received a dose of 50 µg/kg body weight (bw)/day (maximum 4 mg/day) by subcutaneous injection. In case of unacceptable side effects the dose was to be lowered by 25% to 38 µg/kg/day for the rest of the study.

### Measurements

The GH exposure of each subject was recorded at baseline and on Days 7–8 (also on Days 8–9 for patients only) by measuring GH concentration every 30 min (± 10 min) for 24 h, starting immediately after dosing at 20·00 h. GH exposure was determined as the AUC from the 24-h GH profile. The primary end-point was the GH exposure at steady-state (AUC_0–24 h_) on Days 7–8. Determination of GH in serum samples was performed using an enzyme-linked immunosorbentassay (ELISA) method [kit 10–1900; Diagnostic Systems Laboratories (DSL) Inc, Webster, TX] with an intra- and interassay coefficient of variation (CV) of 4·0% and 6·5%, respectively. AUC_0–24 h_ was calculated by the trapezoidal rule.

Secondary efficacy variables included PK and PD assessments. Fasting blood samples for analysis of PD variables in serum at screening and Day 8 were taken after an overnight fast. ELISA methods were used for all analyses (IGF-I: kit 10–5600, intra- and interassay CV 6·0% and 6·7%, respectively; IGFBP-I: kit 10–7800, intra- and interassay CV 2·9% and 6·9%, respectively; IGFBP-III: kit 10–6600, intra- and interassay CV 8·8% and 10·0%, respectively; GHBP: kit 10–48100, intra- and interassay CV 4·5% and 6·6%, respectively; kits all supplied by DSL Inc). The molar ratio for IGF-I/IGFBP-III was also calculated using the following equation:

(IGF-I/7·5)/(IGFBP-III/30·5)

The safety assessments included routine haematology and clinical chemistry, measured at baseline and on Day 8, and adverse events (AE), which were monitored daily during the study.

### Statistical analysis

The primary analysis was based on a 90% CI of the ratio (patients : healthy subjects) of the AUC_0–24 h_ of GH at steady-state (Days 7–8). With 10 subjects per group, a statistical power of more than 80% was obtained, enabling us to claim that the 90% CI lies embedded in the interval 67–150%. This interval was considered to be sufficiently narrow to allow us to evaluate whether or not renal disease had a clinically significant effect. The statistical power calculation was made based on the hypothesis that there was no difference between the mean AUC_0–24 h_ in the two populations and a CV for AUC_0–24 h_ of 30% (data on file, Novo Nordisk^29^).

All subjects carried out trial procedures according to the protocol and were included in the primary analysis. For the primary analysis, a linear normal model (ancova) for the logarithmically transformed values of AUC_0–24 h_ was used. The model included effects of subject group, daily dose/kg bw, and gender. From the model, the difference in means of the log-transformed values was estimated together with 90% CI and these estimates were then exponentially transformed in order to obtain estimates of the patients : healthy subjects ratio and 90% CI.

Secondary PK end-points AUC_0–12 h_, AUC_18–24 h_, AUC_0–∞_, and C_max_ at steady-state were analysed as described for the primary end-point, as were total body clearance (Cl/f) and *t*_1/2,z_, except that these end-points were considered to be dose-independent, thus the dose factor was omitted from the models. Time to reach maximum GH concentration (*t*_max_) was analysed using the nonparametric Kruskal–Wallis test based on Wilcoxon scores and the median difference between the two subgroups was estimated together with a 90% CI using the Hodges–Lehmann estimator.

Analysis of the PD end-points IGF-I and IGFBP-III was performed on the standard deviation score values, including an adjustment for gender. The following formula was used to calculate the SD score values:

SD score = *X* – [(*H* + *L*)/2]/(*H* – *L*)/4

where *X* corresponds to the biomarker and *L* and *H* are the respective low and high laboratory references (DSL Inc). Change from baseline to steady-state for all PD end-points was analysed between subject groups by an ancova, including subject group as fixed effect and the baseline assessment as covariate.

No statistical analyses were performed on the safety end-points.

The Statistical Analysis System software package (version 8·2; SAS Institute, Cary, NC) was used to analyse results. A *P*-value < 0·05 was taken to indicate significance and all statistical tests were two-sided.

## Results

Trial subjects were recruited between June and November 2005. Of the 21 subjects allocated to treatment, a total of 20 (10 patients and 10 healthy subjects) completed the trial. One patient was withdrawn by the sponsor on Day 8 due to a shunt obstruction (evaluated as unlikely to be related to trial product by both investigator and sponsor), but was included in the primary outcome analysis on Days 7–8. There were two protocol deviations affecting six subjects: the follow-up visit for the withdrawn patient was performed later than planned and venous sampling for five subjects began later than 20·10 h (planned 20·00 h ± 10 min) for some visits. These were considered not to have had any bearing on the trial results.

At baseline, IGFBP-I and IGFBP-III values were significantly higher in patients compared with healthy subjects at baseline (Table 2), whereas GHBP values in healthy subjects at baseline were almost double those in patients (*P* = 0·01). There was no significant difference in IGF-I values or IGF-I/IGFBP-III molar ratio between patients and healthy subjects.

**Table 2:** Baseline endocrine parameters in the patients and healthy subjects

Variable	Patients (*N* = 11)	Healthy subjects (*N* = 10)	*P*-value (95% CI)
PTH (pmol/l)	39·9 ± 34·0 (4·0–109·1)	3·8 ± 1·1 (2·4–6·1)	ND
AUC_18–24 h_(µg h/l)	2·86 ± 3·91	1·09 ± 1·43	ND
IGF-I SD score	–0·6 ± 0·9 (–1·7–0·9)	–1·0 ± 0·9 (–1·8–1·1)	0·32 (–0·42; 1·24)
IGF-I (ng/ml)	210 ± 76 (117–322)	178 ± 76 (111–362)	0·30 (0·85; 1·66)
IGFBP-I (ng/ml)	67·4 ± 42·3 (6·3–126·3)	26·8 ± 28·3 (6·4–85·4)	0·03 (1·08; 6·19)
IGFBP-III SD score	2·2 ± 1·5 (–0·6–4·7)	0·4 ± 0·8 (–1·3–1·5)	0·004 (0·63; 2·90)
IGFBP-III (ng/ml)	5025 ± 1038 (3067–6654)	3708 ± 367 (3208–4211)	0·002 (1·13; 1·57)
GHBP (pmol/l)	993 ± 610 (186–1972)	1743 ± 512 (1134–2586)	0·01 (0·27; 0·82)
IGF-I/IGFBP-III molar ratio	0·2 ± 0 (0·1–0·2)	0·2 ± 0·1 (0·1–0·4)	0·37 (0·68; 1·16)

Values are the mean ± SD (range), except for AUC_18–24 h_(mean ± SD).

PTH, parathyroid hormone; ND, not done; IGF-, insulin-like growth factor-; IGFBP-, insulin-like growth factor binding protein-; SD, standard deviation.

### Primary end-point

The geometric mean GH AUC_0–24 h_ at steady-state (Days 7–8) was two-thirds greater for patients than for healthy subjects (Table 3). A difference between patients and healthy subjects was indicated by the fact that the 90% CI for the estimated patient : healthy subject ratio did not fall within the acceptance interval (0·67–1·50).

**Table 3:** Primary and secondary pharmacokinetic (PK) efficacy endpoints at steady-state (Days 7–8)

Variable	Patients (*N* = 11)	Healthy subjects (*N* = 10)	Patients : healthy subjects ratio	90% CI
AUC_0–24 h_ (µg h/l)*	387·91 ± 134·13	225·35 ± 59·63	1·71	1·40; 2·07
AUC_0–12 h_ (µg h/l)	360·16 ± 134·04	194·00 ± 57·42	1·84	1·49; 2·26
AUC_18–24 h_ (µg h/l)	3·03 ± 2·71	6·37 ± 4·21	0·48	0·26; 0·88
AUC_0–8_ (µg h/l)	388·90 ± 134·07	228·53 ± 60·45	1·69	1·39; 2·05
C_max_(µg/l)	55·15 ± 26·87	27·29 ± 10·90	1·99	1·53; 2·59
*t*_max_(h)	5·00 (3·00; 6·50)	4·50 (3·50; 9·00)	N/A	–1·0; 1·0
Cl/f (l/h)	9·64 ± 3·61	16·45 ± 6·11	0·59	0·45; 0·76
*t*_1/2,z_ (h)†	2·28 ± 0·43	3·23 ± 0·75	0·71	0·60; 0·83

Values are the geometric mean ± SD, except for *t*_max_[median (minimum; maximum)].

* Primary end-point;

† Terminal half-life; N/A, not applicable.

### Secondary PK end-points

**Fig. 2:**
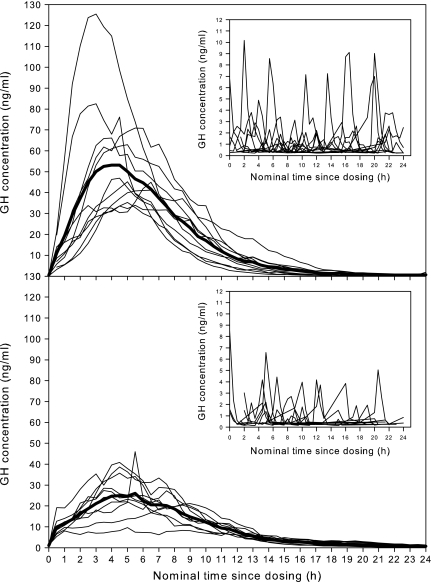
Individual (narrow lines) and mean (thick lines) GH profiles for patients (top) and healthy subjects (bottom) on Days 7–8. Inserts show individual profiles on Day 1.

The GH AUC_0–12 h_ geometric mean at steady-state for patients was almost double that for healthy subjects (Table 3). Based on the 90% CI, a difference between patients and healthy subjects was indicated for this and all other secondary PK end-points. The geometric mean GH AUC_18–24 h_ at steady-state for healthy subjects was double that for patients. Individual/mean GH profiles on Days 7–8 are shown in Fig. 2. Of particular note is the fact that GH concentrations for both patients and healthy subjects were comparable with baseline values by about 20–22 h, demonstrating that administered rhGH does not seem to be retained in the circulation of patients with ESRD. Moreover, the geometric mean GH AUC_18–24 h_ steady-state for both patients and healthy subjects (Table 3) was about the same as the baseline mean values (Table 2). Individual GH profiles (not shown) for the period 18–24 h on Days 7–8 were relatively flat compared to the peaks obtained on Day 1 for all subjects (particularly noticeable for the patient group) (Fig. 2, inserts), demonstrating that administration of rhGH appeared to suppress endogenous GH production.

The geometric mean GH AUC_0–∞_ steady-state for patients was two-thirds greater than that for healthy subjects. The C_max_ geometric mean for patients at steady-state was double that for healthy subjects. With regard to *t*_max_, the median values for patients and healthy subjects were approximately the same and no significant difference was found between patients and healthy subjects (*P* = 0·8). Individual GH profiles (Fig. 2) also demonstrated the higher GH concentration (C_max_ and AUC_0–24 h_) measured for patients compared to healthy subjects, and also that *t*_max_ for both groups was reached in 4–5 h (range 3–9 h) after dosing.

The geometric mean of Cl/f at steady-state for healthy subjects was two-thirds greater than that for patients. Similarly, the geometric mean of *t*_1/2,z_ for healthy subjects was greater (1·4 times) than that for patients.

In patients only, GH variables on Days 7–8 were compared with the same period on Days 8–9, which included a dialysis session (data not shown). There appeared to be no difference between values on Days 7–8 and Days 8–9 for any secondary PK end-point, except for AUC_18–24 h_ and *t*_max_(Days 7–8 mean values were greater than Days 8–9 values) and C_max_(Days 8–9 values were greater).

### Secondary PD end-points

The mean PD data showing change from baseline to steady-state are presented in Table 4. Only IGFBP-III and associated SD score showed a significant difference (*P* = 0·001) between patients and healthy subjects. IGF-I SD score, and to a lesser extent IGFBP-III SD score, increased in both groups compared with baseline, whereas GHBP, and to a lesser extent IGFBP-I, decreased. The IGF-I/IGFBP-III molar ratio increased slightly for both groups, but there was no significant difference between groups. It should be noted that data for all biomarkers, except IGFBP-III, were highly variable, as depicted in the SDs, particularly in the patient group.

**Table 4:** Secondary pharmacodynamic (PD) efficacy end-points at steady-state (Days 7–8), change from baseline

Variable, change from baseline	Patients (*N* = 11)	Healthy subjects (*N* = 10)	Patients – healthy subjects difference	*P*-value (95% CI)
IGF-I SD score	9·3 ± 4·5	7·7 ± 1·5	0·84	0·55 (–2·08; 3·76)
IGF-I (ng/ml)	756 ± 361	630 ± 98	74·4	0·51 (–159; 308)
IGFBP-I (ng/ml)	–31·6 ± 39·6	–18·7 ± 27·8	12·7	0·36 (–15·6; 41·0)
IGFBP-III SD score	3·4 ± 0·6	2·2 ± 0·5	1·22	0·001 (0·58; 1·86)
IGFBP-III (ng/ml)	2615 ± 467	1693 ± 370	1029	0·001 (505; 1553)
GHBP (pmol/l)	–74·1 ± 167·2	–195 ± 134·9	69·6	0·40 (–100; 239)
IGF-I/IGFBP-III molar ratio	0·3 ± 0·2	0·4 ± 0·0	–0·07	0·20 (–0·18; 0·04)

Values are the mean ± SD. IGF-, insulin-like growth factor-; IGFBP-, insulin-like growth factor binding protein-; SD, standard deviation.

There was no significant difference in any of the PD biomarker values on Days 7–8 compared with Days 8–9 in ESRD patients (data not shown).

### Safety

No major safety issues were identified during the trial. The most common adverse events were headaches (13 events, reported equally between patients and healthy subjects) and peripheral oedemas (six events, all but one in healthy subjects). During the trial, two subjects had the rhGH dose reduced. One patient experienced a severe adverse event (vomiting), which lasted a day and one healthy subject had a headache that lasted for 8 days. These events were considered probably related to trial product and the dose of rhGH was reduced on Day 7 and Day 5, respectively.

## Discussion

In this study, treatment with GH did result in higher exposure in patients with ESRD than in healthy subjects on Days 7–8, as demonstrated by the markedly greater AUC_0–24 h_ (primary end-point), AUC_0–12 h_ and C_max_. However, as the individual subjects profiles were back to baseline by 20–22 h, it is concluded that no overall accumulation of GH occurs. Results of this study were in agreement with previous studies which showed a greater AUC^25^ and a reduced metabolic clearance rate^23^–^25^ in patients with chronic renal failure compared with in healthy controls, following intravenous administration of GH. The fractional clearance rate Cl/f in our study was also lower in ESRD patients than in healthy subjects. Taken together, results demonstrate an impaired clearance of GH in patients with ESRD, supporting that the kidney plays an important role in GH elimination.^23^–^25^

In the current study, the terminal half-life *t*_1/2,z_ was shorter for patients compared with for healthy subjects, resulting in AUC_18–24 h_for ESRD patients being lower than for healthy subjects. This, combined with the fact that GH concentrations 20–22 h after dosing on Day 8 were similar to baseline values for both groups, indicates that GH did not remain in the circulation of ESRD patients longer than in healthy individuals, as had been considered a possibility at the outset of the study.^23^,^25^,^26^ Moreover, the return to baseline values for both treatment groups suggested that the difference in AUC_18–24 h_ between groups was likely to be of no clinical significance.

The metabolic half-life of GH has been reported to vary between about 7–50 min in healthy subjects, and it is significantly longer in patients with chronic renal failure.^23^–^25^ In this study, on the other hand, *t*_1/2,z_ for healthy individuals was 03·23 h, in agreement with the 2–4 h observed in previous studies of the PK of subcutaneous GH administration in healthy males.^30^,^31^ PK parameters for GH have been shown to vary depending on whether the administration method used is transcutaneous or subcutaneous, and it has been demonstrated that GH kinetics after subcutaneous administration have a ‘flip-flop’ characteristic, whereby the absorption rate approximates the rate of elimination.^32^ Results of our study support that conclusion. Patients with ESRD had a shorter *t*_1/2,z_ of 02·28 h, reflecting an apparent higher absorption rate for patients compared with healthy volunteers. The higher AUC_0–24 h_ in patients compared with in healthy subjects therefore may be explained by a lower overall clearance, whereas the higher C_max_ may be explained by a combination of the lower clearance and a faster absorption (suggested by the faster terminal slope). Additionally, the lower AUC_18–24 h_ for ESRD patients partly reflects a faster absorption compared with in healthy individuals.

The wide variation in the GH half-lives reported in the literature has been explained by the use of different methodologies, and interference by the secretion of endogenous GH.^23^,^25^ A limitation of the current study therefore is that endogenous GH secretion was not suppressed. Nevertheless, our results were in agreement with other studies, and the fact that individual GH profiles at 18–24 h on Days 7–8 were relatively flat compared to those obtained on Day 1 suggests that GH administration did suppress endogenous GH production to some extent.

The reduced clearance observed for ESRD patients in the current study (58% of that for healthy individuals) is in agreement with other studies which have demonstrated a reduction in the metabolic clearance rate (MCR) of GH of about 50% for patients with chronic renal failure, and commensurate with a GH plasma half-life double that of healthy controls.^23^–^25^ GH appears to be eliminated in humans by a biocompartmental model,^25^,^33^ with unbound GH in a central compartment and GHBPs and bound GH in a peripheral compartment. In healthy individuals, the kidney accounts for about 50% of the total MCR,^23^ and it is less than this in patients with renal disease,^24^ whereas extrarenal elimination (mostly via the liver) does not seem to differ significantly between the two groups.^23^ However, the situation is made more complex by the fact that GH also exists (reversibly) bound to GHBP, which is decreased in ESRD patients; the relative amounts of bound GH will most likely also correlate with the GH elimination rate.

No major safety concerns were raised with this trial. The high mean C_max_ (double that for healthy controls) and greater AUC_0–24 h_ observed for patients with ESRD might be considered to have the potential to adversely influence the safety profile of rhGH. However, this seems not to be the case as GH appeared to be well tolerated generally and no differences in the safety profiles between the two groups were observed. The most common adverse events were headache and peripheral oedema, but these were not unexpected and neither occurred with greater incidence in the ESRD patient group. It is known that short-term rhGH administration can lead to fluid retention.^34^ In this trial, most events of oedema occurred in healthy subjects, probably because body fluid control is strictly regulated during dialysis treatment. One patient and one healthy subject had a GH dose reduction. The rhGH dose chosen was the highest dose employed in a previous trial of subcutaneous GH treatment in 139 patients with ESRD (37 dosed with 50 µg/kg bw/day, as in this trial), where GH treatment was found to be safe.^21^

With respect to standard GH biomarkers at steady-state, only IGFBP-III showed a significant difference between patients and healthy subjects after GH treatment and was greater for patients. Patients with ESRD are often resistant to GH and have a diminished rate of secretion of IGF-I.^12^ In concordance with previous trials, this study demonstrated an increase in IGF-I and IGF-I SD score in patients with ESRD after treatment with GH.^13^,^15^–^17^,^20^ There was a similar increase in healthy subjects, and no significant difference between the two groups was observed.^13^,^15^,^16^,^20^ Concerns have been raised regarding the potential risk of cancer associated with increased IGF-I as a result of GH exposure, and the molar ratio of IGF-I and IGFBP-III may be important in determining the relative risk of malignancy.^35^ In the current study, although IGFBP-III was significantly greater for patients compared with healthy subjects, as has been observed previously during GH treatment of patients receiving haemodialysis,^22^ both IGF-I and the IGF-I/IGFBP-III molar ratio increased for both patients and healthy subjects, as was also reported previously,^14^,^15^ and there was no significant difference between groups. A possible role for IGF-I monitoring has been suggested to avoid morbidities associated with IGF-I excess, as well as deficiency.^35^ It should be noted that size-exclusion chromatography (to exclude any immunoreactive fragments of IGFBP-III formed as a result of IGFBP-III protease activity) was not performed in this study; therefore, concentrations of IGFBP-III may have been overestimated.

In agreement with previous reports,^23^,^36^,^37^ the baseline concentrations of IGFBP-III and IGFBP-III SD score were significantly elevated in patients with ESRD. IGFBP-I was also significantly greater in ESRD patients at baseline, as has been previously reported,^13^ whereas baseline GHBP was significantly reduced. Previous investigations of patients with chronic renal failure have also observed reduced concentration of GHBP,^24^,^38^ which may indicate decreased expression of the GH receptor in target tissues, and hence diminished responsiveness to GH in renal failure. The lower concentrations of GHBP may also have contributed to the observed reduction in plasma clearance (and hence increase in AUC) in the ESRD patients compared with healthy subjects. A PK study of intravenous GH administration found a strong positive correlation between baseline GHBP concentration and MCR at physiological GH concentrations.^39^ In addition, the reduced GH elimination rate observed in GH-deficient patients in another study was explained by lower levels of GHBP, possibly reflecting a reduced GH receptor density and hence clearance.^40^

In contrast to a study of patients with chronic renal failure undergoing dialysis,^13^ baseline IGF-I was not significantly higher in patients compared with healthy controls in the present study. In general, patients with ESRD appear to exhibit normal or high concentrations of IGF-I and considerably elevated concentrations of IGFBP-I and IGFBP-III, which increase with declining renal function,^13^ demonstrating that the GH–IGF-I axis is affected on several levels.

There was no marked effect of dialysis on GH PK and PD parameters, assessed in patients only on Days 8–9. The differences observed in AUC_18–24 h_, *t*_max_and C_max_ between steady-state and dialysis were judged to be of no clinical significance.

To date, a number of trials have demonstrated a benefit of GH treatment in patients with ESRD. Major adverse events have not been reported and GH does not appear to accumulate in the circulation of ESRD patients. However, further trials are needed to assess the ultimate effects of GH treatment on morbidity and mortality.
